# Efficacy of personalized repetitive transcranial magnetic stimulation based on functional reserve to enhance ambulatory function in patients with Parkinson’s disease: study protocol for a randomized controlled trial

**DOI:** 10.1186/s13063-024-08385-2

**Published:** 2024-08-16

**Authors:** Seo Jung Yun, Ho Seok Lee, Dae Hyun Kim, Sun Im, Yeun Jie Yoo, Na Young Kim, Jungsoo Lee, Donghyeon Kim, Hae-Yeon Park, Mi-Jeong Yoon, Young Seok Kim, Won Hyuk Chang, Han Gil Seo

**Affiliations:** 1 Department of Rehabilitation Medicine, Seoul National University Hospital, Seoul, Republic of Korea; 2 Department of Rehabilitation Medicine, Seoul National University College of Medicine, Seoul, Republic of Korea; 3 Department of Human Systems Medicine, Seoul National University College of Medicine, Seoul, Republic of Korea; 4 Department of Physical and Rehabilitation Medicine, Center for Prevention and Rehabilitation, Heart Vascular Stroke Institute, Samsung Medical Center, Sungkyunkwan University School of Medicine, Seoul, Republic of Korea; 5 Department of Rehabilitation Medicine, Bucheon St. Mary’s Hospital, College of Medicine, The Catholic University of Korea, Seoul, Republic of Korea; 6 Department of Rehabilitation Medicine, St. Vincent’s Hospital, College of Medicine, The Catholic University of Korea, Seoul, Republic of Korea; 7 Department of Rehabilitation Medicine, Yongin Severance Hospital, Yonsei University College of Medicine, Yongin, Republic of Korea; 8 Department of Medical IT Convergence Engineering, Kumoh National Institute of Technology, Gumi, Republic of Korea; 9 Research Institute, NEUROPHET Inc, Seoul, Republic of Korea; 10 Department of Health Science and Technology, Department of Medical Device Management and Research, SAIHST, Sungkyunkwan University, Seoul, Republic of Korea

**Keywords:** Functional reserve, Gait, Repetitive transcranial magnetic stimulation, Parkinson’s disease

## Abstract

**Background:**

Repetitive transcranial magnetic stimulation (rTMS) is one of the non-invasive brain stimulations that modulate cortical excitability through magnetic pulses. However, the effects of rTMS on Parkinson’s disease (PD) have yielded mixed results, influenced by factors including various rTMS stimulation parameters as well as the clinical characteristics of patients with PD. There is no clear evidence regarding which patients should be applied with which parameters of rTMS. The study aims to investigate the efficacy and safety of personalized rTMS in patients with PD, focusing on individual functional reserves to improve ambulatory function.

**Methods:**

This is a prospective, exploratory, multi-center, single-blind, parallel-group, randomized controlled trial. Sixty patients with PD will be recruited for this study. This study comprises two sub-studies, each structured as a two-arm trial. Participants are classified into sub-studies based on their functional reserves for ambulatory function, into either the motor or cognitive priority group. The Timed-Up and Go (TUG) test is employed under both single and cognitive dual-task conditions (serial 3 subtraction). The motor dual-task effect, using stride length, and the cognitive dual-task effect, using the correct response rate of subtraction, are calculated. In the motor priority group, high-frequency rTMS targets the primary motor cortex of the lower limb, whereas the cognitive priority group receives rTMS over the left dorsolateral prefrontal cortex. The active comparator for each sub-study is bilateral rTMS of the primary motor cortex of the upper limb. Over 4 weeks, the participants will undergo 10 rTMS sessions, with evaluations conducted pre-intervention, mid-intervention, immediately post-intervention, and at 2-month follow-up. The primary outcome is a change in TUG time between the pre- and immediate post-intervention evaluations. The secondary outcome variables are the TUG under cognitive dual-task conditions, Movement Disorder Society-Unified Parkinson’s Disease Rating Scale Part III, New Freezing of Gait Questionnaire, Digit Span, trail-making test, transcranial magnetic stimulation-induced motor-evoked potentials, diffusion tensor imaging, and resting state functional magnetic resonance imaging.

**Discussion:**

The study will reveal the effect of personalized rTMS based on functional reserve compared to the conventional rTMS approach in PD. Furthermore, the findings of this study may provide empirical evidence for an rTMS protocol tailored to individual functional reserves to enhance ambulatory function in patients with PD.

**Trial registration:**

ClinicalTrials.gov NCT06350617. Registered on 5 April 2024.

**Supplementary Information:**

The online version contains supplementary material available at 10.1186/s13063-024-08385-2.

## Introduction

### Background and rationale {6a}

Parkinson’s disease (PD) is the second most common neurodegenerative disorder, characterized by cardinal symptoms including resting tremor, bradykinesia, and rigidity, alongside non-motor symptoms such as cognitive impairment, depression, and autonomic dysfunction [1]. The current gold standard treatment for PD involves dopaminergic medications, which alleviate symptoms without impeding disease progression [2]. However, prolonged use of these medications may lead to complications such as levodopa-induced dyskinesia [3]. Surgical interventions, such as deep brain stimulation of the subthalamic nucleus or globus pallidus interna, are available for some patients, although eligibility is limited [4]. The global prevalence of PD is increasing, which is attributable to the rapid expansion of the aging population [5]. Therefore, ongoing research into disease-modifying therapies is necessary to manage symptoms and slow disease progression.

Repetitive transcranial magnetic stimulation (rTMS) is a non-invasive brain stimulation that modulates cortical excitability through magnetic pulses [6]. In PD, rTMS has been employed to enhance motor and gait function by targeting areas such as the primary motor cortex (M1), dorsolateral prefrontal cortex (DLPFC), supplementary motor area (SMA), and cerebellum using various stimulation parameters [7–9]. High-frequency rTMS over the M1, DLPFC, and SMA has demonstrated positive effects on overall motor symptoms in PD [9]. While an increase in dopamine secretion in the basal ganglia via the cortico-striatal pathways may contribute to improvements in motor function, the precise underlying mechanism of rTMS in PD remains to be elucidated [10, 11]. Conversely, intermittent theta burst stimulation of the M1 and DLPFC, or high-frequency rTMS of the bilateral motor cortex, does not significantly benefit motor function in PD [12, 13]. Likewise, research on the application of varied rTMS methodologies to enhance motor function in PD has been conducted, yielding diverse outcomes based on these rTMS approaches (Table 1).

**Table 1:** Characteristics of randomized controlled trials applying high-frequency repetitive transcranial magnetic stimulation to improve motor function in Parkinson’s disease

Study	Site	Stimulation order	Frequency	Intensity	Session	Sample size	Control	Motor outcome
Benninger, 2012 [12]	Bilateral M1-UL	Alternating	50	AMT 80%	8	26	Sham (13 vs 13)	10MWT ( −) UPDRS III ( −)
Brys, 2016 [14]	Bilateral M1-UL	Sequential (L → R)	10	RMT	10	29	Sham on DLPFC (14 vs 15)	UPDRS III ( +)
Khedr, 2019 [15]	Bilateral M1-UL	Sequential (R → L)	20	RMT 90%	10	30	Sham (19 vs 11)	UPDRS III ( +)
Spagnolo, 2021 [16]	Bilateral M1-UL	More affected, but bilaterally	10	RMT 90%	12	20	Sham on PFC (20 vs 20)	UPDRS III ( +)
Makkos, 2016 [17]	Bilateral M1-UL	Sequential (R → L)	5	RMT 90%	10	23	Sham on PFC (21 vs 20)	TUG ( −) UPDRS III ( +) HY ( −)
Yokoe, 2018 [18]	Bilateral M1-UL	Sequential (less affected → more affected)	10	RMT 100%	1	19	Crossover	UPDRS III ( +)
Li, 2020 [19]	M1-UL	Contralateral where feeling pain bilateral pain → left M1	20	RMT 80%	5	48	Sham (24 vs 24)	UPDRS III ( +)
Chang, 2016 [20]	M1-LL	Dominant hemisphere	10	RMT 90%	5	8	Crossover (2 weeks wash-out)	TUG ( +) UPDRS III ( +)
Kim, 2015 [21]	M1-LL	Dominant hemisphere	10	RMT 90%	5	17	Crossover (2 weeks wash-out)	TUG ( +) UPDRS III ( +)
Maruo, 2013 [22]	Bilateral M1-LL	Sequential (more affected → less affected)	10	RMT 100%	3	21	Crossover	10MWT ( +) UPDRS III ( +) Finger tapping ( +)
Chung, 2020 [23]	Bilateral M1-LL	Sequential (more affected → less affected)	25	RMT 80%	12	33	Sham (17 vs 16)	10MWT ( +) 7 m TUG ( +) 7 m TUG-Cog ( +) UPDRS III ( +)
Yang, 2013 [24]	M1-LL	Contralaterally to the more affected side	5	RMT 100%	12	20	Sham (10 vs 10)	10MWT-comfortable speed ( −) 10MWT-fast speed ( +) TUG ( +)
Khedr, 2003 [25]	M1-LL, bilateral M1-UL	Sequential (R → L)	5	MT 120% for hand	10	36	Sham (19 vs 17)	Gait speed ( +) UPDRS III ( +)
Brys, 2016 [14]	Left DLPFC	-	10	RMT	10	27	Sham on DLPFC (12 vs 15)	UPDRS III ( −)
Pal, 2010 [26]	Left DLPFC	-	5	RMT 90%	10	22	Sham (12 vs 10)	UPDRS III ( −)
del Olmo, 2007 [27]	DLPFC	Contralaterally to the more affected side	10	RMT 90%	10	13	Sham (8 vs 5)	Gait ( −) Finger tapping ( +)
Yokoe, 2018 [18]	DLPFC	Sequential (less affected → more affected)	10	RMT 100%	1	19	Crossover	UPDRS III ( −)
Brys, 2016 [14]	M1-UL and left DLPFC	Left DLFPC → bilateral M1 (L → R)	10	RMT	10	35	Sham on DLPFC (20 vs 15)	UPDRS III ( −)
Lomarev, 2006 [28]	Bilateral M1-UL and bilateral DLPFC	L → R	25	RMT 100%	8	16	Sham (7 vs 9)	10MWT ( +)
Spagnolo, 2021 [16]	M1-UL and PFC	M1-UL → PFC more affected, but bilaterally	10	(M1) RMT 90% (PFC) RMT 100%	12	19	Sham on PFC (20 vs 20)	UPDRS III ( +)

*M1-UL* Primary motor cortex in the upper limb, *AMT* Active motor threshold, *10MWT* 10-m walk test, *UPDRS* Unified Parkinson’s Disease Rating Scale, *L* Left, *R* Right, *RMT* Resting motor threshold, *DLPFC* Dorsolateral prefrontal cortex, *PFC* Prefrontal cortex, *TUG* Timed-Up and Go, *HY* Hoehn and Yahr scale, *M1-LL* Primary motor cortex in the lower limb, *TUG-Cog* Timed-Up and Go under cognitive dual-task condition, *MT* Motor threshold, *M1* Primary motor cortex

rTMS applied to the DLPFC also alleviates the non-motor symptoms of PD, such as depression and cognitive impairment [29]. Multiple sessions of high-frequency rTMS targeted at the DLPFC could enhance executive function in PD [26, 30]. In contrast, high-frequency rTMS of the left DLPFC leads to a non-significant reduction in depressive mood among patients with PD [14]. The exact rationale behind these varied responses in PD to rTMS treatment remains speculative, highlighting the need for further research to uncover the underlying mechanisms thereof. The effects of rTMS in PD have yielded mixed results, influenced by factors including the rTMS stimulation site, frequency, intensity, total number of pulses, and the number of sessions, as well as clinical subtypes of patients with PD [15, 31]. Furthermore, variability in the extent of basal ganglia damage among patients presents challenges in achieving consistent outcomes with standardized rTMS treatment protocols. A personalized rTMS approach targeting heterogeneous patient’ characteristics, including the presence of tremors, freezing, motor fluctuation, and dyskinesia may be necessary to maximize the effect of rTMS [7].

Recently, the concept of functional reserve has been proposed for patients with PD [32]. This concept emerged from the manifestation of inconsistent symptoms in patients with similar degrees of nigrostriatal dopaminergic deficits from dopamine transporter (DAT) imaging [23]. In a study assessing motor functional reserve using the Movement Disorder Society-Unified Parkinson’s Disease Rating Scale (MDS-UPDRS) Part III and DAT, functional connectivity analysis using resting-state functional magnetic resonance imaging (rsfMRI) confirmed that motor functional reserve was associated with the functional connectivity of brain networks in PD, involving structures such as the basal ganglia and inferior frontal lobe. Another study found an association between motor functional reserve in PD and striatal volume [33]. Hence, it is conceivable that the motor function reserve in PD is related to the neural network connectivity in the basal ganglia and frontal lobe. Additionally, the concept of resilience in patients with PD includes the cognitive functional reserve. Therefore, targeting functional improvement based on individual functional reserve, which encompasses motor and cognitive functions, as well as the degree of structural damage to the brain, is necessary for the management of PD [32].

Single-and dual-task assessments are primarily utilized to discern functional reserves in patients with PD [34]. In the early stages of PD, there is a reduction in gait automaticity due to impairment of the sensorimotor circuit of the basal ganglia [35]. Therefore, patients with PD compensate by engaging in goal-directed networks to perform dual-tasks, instead of relying on the negatively affected habitual control pathway [36]. Discrepancies in performance between single- and dual-tasks could shed light on the underlying functional adaptation mechanisms, whether motor or cognitive. One study compared brain activity patterns in groups that focused on motor and cognitive functions, revealing increased activity in the prefrontal and parietal cortex of the cerebrum among the participants in the cognitive function-focused group [37]. This suggests that patients who prioritize cognitive function may leverage prefrontal cortex functions such as coordination, concentration, and execution in their efforts for behavioral enhancement. In such patients, enhancing the cognitive network may prove to be a more efficient strategy to improve gait and daily functional abilities than attempting to restore already lost motor functions. In PD, a higher cognitive reserve is associated with a lower overall cognitive impairment and reduced severity of motor symptoms [38, 39]. Motor reserves in PD explain the variations in motor deficits observed among patients despite having comparable levels of striatal dopamine depletion [40]. These concepts are expected to provide significant insights into the implementation of personalized rTMS interventions aimed at enhancing resilience against neurodegenerative changes.

Gait impairment in PD is one of the most disabling conditions and is associated with an increased risk of falls, reduced independence, and diminished quality of life [41, 42]. Improvements in simple motor symptoms, while beneficial, may not directly translate into functional enhancements that offer immediate benefits to patients with PD. The Timed-Up and Go (TUG) test in patients with PD is as an invaluable instrument to evaluate transitions, balance, and gait. In addition, when combined with gait analysis under both single- and dual-task conditions, the TUG test facilitates the quantitative assessment of overall gait function [42].

Hence, the variability in symptom improvement among patients with PD could be attributed to individual differences in motor and cognitive functional reserves. Consequently, designing effective rTMS treatment protocols necessitates a thorough assessment of each patient’s functional impairment and reserve capacity. Incorporating the concepts of motor and cognitive reserves into treatment planning allows for the tailoring of individualized rTMS protocols, optimizing treatment outcomes for patients with PD.

### Objectives {7}

The study aims to investigate the efficacy and safety of personalized rTMS in patients with PD, focusing on individual functional reserves to improve ambulatory function. The participants are categorized into motor or cognitive priority groups based on their functional reserve determined through single- and dual-task assessments, and specific rTMS strategies implemented that reflect their unique characteristics.

#### Trial design {8}

This is a prospective, exploratory, multi-center, single-blind, parallel-group, randomized controlled trial.

### Methods: participants, interventions, and outcomes

#### Study setting {9}

The study will be conducted across five tertiary hospitals in the Republic of Korea, including Samsung Medical Center, Seoul National University Hospital, and Yongin Severance Hospital as well as Bucheon St. Mary’s Hospital and St. Vincent’s Hospital, both of which are branches of the Catholic Medical Center. The study will be performed in accordance with the principles of Good Clinical Practice and the Declaration of Helsinki.

#### Eligibility criteria {10}

The inclusion criteria are as follows:

- Patients clinically diagnosed with idiopathic PD following the UK Parkinson’s Disease Society Brain Bank Diagnostic Criteria
- Modified Hoehn and Yahr scale 2 to 4
- Patients capable of walking on level ground without the use of a gait aid
- Aged ≥ 50 years
- Patients who have provided informed consent and voluntarily signed the written consent form for participation in the study

The exclusion criteria include:

- Patients with contraindications for rTMS, a history of epilepsy, any metal inserted into the head, or who had undergone cranial surgery
- Patients exhibiting cognitive impairment based on the Korean-Montreal Cognitive Assessment test, with the following cutoff scores [43]:
  - < 7 points: Illiterate,
  - < 13 points: Education duration 0.5–3 years,
  - < 16 points: Education duration of 4–6 years,
  - < 19 points: Education duration of 7–9 years, and
  - < 20 points: Education duration 10 years or more

- Concurrent major neurological conditions, such as spinal cord injury and stroke
- Existing significant psychiatric disorders requiring continuous medication, such as major depressive disorder, schizophrenia, bipolar disorder, or dementia
- Severe dyskinesia or severe on–off phenomenon
- Pregnancy and lactation
- Participants with contraindications for MRI, such as those with implanted devices like pacemakers
- Refuse to participate in the study

#### Who will take informed consent? {26a}

The Korean Pharmacists Act mandates that a physician serving as the investigator should obtain informed consent from prospective clinical trial participants or their authorized representatives. Investigators are obliged to elucidate the contents of the finalized Institutional Review Board (IRB)-approved informed consent form to ensure a comprehensive understanding among potential research participants. This includes explaining the purpose of the study, the benefits, and harms involved and providing clear channels to contact both the investigator and the IRB for any questions that may arise during participation. Following consent acquisition, the investigator should promptly provide the participants with a copy of the consent form.

#### Additional consent provisions for collection and use of participant data and biological specimens {26b}

Not applicable. No biological specimens are collected.

## Interventions

### Explanation for the choice of comparators {6b}

The comparators of both sub-studies are the active control groups. The effectiveness of high-frequency rTMS applied to bilateral primary motor cortex in the upper limb (M1-UL) in patients with PD has been previously demonstrated to enhance general motor performance and alleviate depression and anxiety, aligning with evidence-based rTMS guidelines [9, 44]. Additionally, high-frequency stimulation of M1 has been shown to relieve musculoskeletal pain and improve the quality of life in patients with PD. Therefore, stimulation applied to bilateral M1 regions, as a conventional approach, is chosen as the comparative protocol to validate the superiority of this novel personalized rTMS.

### Intervention description {11a}

This study comprises two sub-studies, each designed as a two-arm trial. The participants are classified into sub-studies based on their functional reserves as follows:

- Motor priority group (sub-study 1): Patients with well-preserved motor function in whom motor skills have a significant impact on overall functioning; and
- Cognitive priority group (sub-study 2): Patients with well-preserved cognitive function and notable impairment in motor function in which cognitive abilities substantially affect overall functioning.

As part of the pre-intervention assessment, the TUG and the TUG under cognitive dual-task condition (TUG-Cog) are administered. The TUG-Cog involves performing the TUG test concurrently with a cognitive task of serially subtracting the number 3. Additionally, serial 3 subtraction as a single cognitive task is performed from a randomly selected number between 80 and 100 for 20 s in the sitting position [34]. For both tasks, the correct response rate for subtraction is calculated as the time spent in seconds divided by the number of correct responses. Task-specific interference is calculated using the equation for dual-task effect (DTE) (Eq. 1) [37]. The motor dual-task effect (mDTE) is computed using stride length and the cognitive dual-task effect (cogDTE) is based on the correct response rate of TUG and TUG-Cog.

1 $$\text{Dual task effect} \left(\%\right)=\frac{(\text{Single task }-\text{ Dual task})}{\text{Single task}} \times 100$$

For evaluation of task prioritization during dual-task conditions, the modified Attention Allocation Index (mAAI) is employed (Eq. 2) [34]. The mAAI is calculated by subtracting the cogDTE from the mDTE, where negative values indicate an attention shift toward the motor task (motor priority), while positive values suggest an attention shift toward the cognitive task (cognitive priority).

2 $$\text{modified Attention Allocation Index }\left(\text{mAAI}\right)=\text{motor dual task effect}\left(\text{mDTE}\right)-\text{cognitive dual task effect }(\text{cogDTE})$$

Patients demonstrating motor priority will undergo high-frequency rTMS over the primary motor cortex in the lower limb (M1-LL), whereas those showing cognitive priority receive high-frequency rTMS over the left DLPFC. Given the substantial impairment of the motor network in the latter group, we envision a heightened reinforcement of compensatory mechanisms utilizing the cognitive network.

In the experimental group of sub-study 1, the more affected M1-LL is stimulated using a double-cone coil with a frequency of 10 Hz and an intensity set at 90% of the participant’s resting motor threshold (RMT) measured in the more affected M1-LL. The RMT in the M1-LL is determined at rest with the tibialis anterior muscle on the more affected side. The RMT is defined as the minimum intensity at which responses of 50 uV or greater are elicited in at least five out of ten trials, measured in a resting state of full relaxation. The more affected side will be determined based on the findings of the MDS-UPDRS Part III performed during at the pre-intervention evaluation. In instances where the assessment does not conclusively identify the more affected side, the onset side of PD symptoms is considered. If the side of onset remains unclear, the non-dominant side is designated as the more affected side. The stimulation protocol consisted of 5 s of stimulation followed by a 25-s rest period, repeated for a total of 20 cycles, resulting in the administration of 1000 stimuli per session. Using this protocol, each session lasts a total of 10 min.

In sub-study 2, the hot spot for the experimental group is the left DLPFC. The left DLPFC will be manually designated based on anatomical landmarks [45]. Once the left DLPFC is determined, the Neurophet tES LAB (Neurophet Inc., Seoul, Republic of Korea) will be used to obtain individual guide information relative to Cz. The software segments each individual’s T1-weighted brain image acquired at pre-intervention evaluation, reconstructs it into a three-dimensional brain model, and provides guidance for coil placement on the scalp. Investigators use the skull caps to apply stimulation at precise locations. The stimulation intensity is set at 100% of the participant’s RMT in the more affected M1-UL. Stimulation frequency, duration, cycles, and total stimuli are the same as those used in the experimental group in sub-study 1.

The bilateral M1-UL is stimulated in the control groups in both sub-studies. The stimulation intensity is adjusted to 90% of the participants’ RMT in the more affected M1-UL. The RMT of the M1-UL is determined at rest with the first dorsal interosseus muscle on the more affected side. Bilateral stimulation is conducted sequentially, starting with the more affected side, followed by the less affected side. A figure-eight coil will be used to stimulate the DLPFC and M1-UL. Other stimulation protocols are consistent with those used in the experimental groups. The stimulation intensity, frequency, duration, and total stimuli were determined based on previous study guidelines [44].

The intervention will employ a Magstim Rapid2 (Magstim Co. Ltd., UK) or MagPro X100 magnetic stimulator (MagVenture, Lucernemarken, Denmark). All participants will undergo 10 rTMS sessions, 2–3 times a week, for 10 min per session over a period of 4 weeks. After completing the initial five sessions within a 2-week period, a mid-intervention evaluation will be conducted. During each session, even-level gait training or treadmill training will be also performed for 10 min immediately after rTMS.

### Criteria for discontinuing or modifying allocated interventions {11b}

The criteria for discontinuing the intervention are as follows:

- Voluntary discontinuation by the participant: Participants have the freedom to withdraw from the trial at any time without providing an explanation, and this will not affect their future treatment.
- Missed follow-up visits.
- Discontinuation due to a significant adverse event or if the participant or legal representative requests termination due to adverse events.
- Based on the investigator’s judgment, the progression of a clinical trial may be deemed unsuitable.
- Significant protocol violation or deviation from inclusion/exclusion criteria during the clinical trial.
- Study adherence < 80%.
  $$\text{Adherence }\left({\%}\right)= \left(\frac{\text{Actual number of rTMS sessions performed}}{\text{Total required rTMS sessions}}\right)\times 100$$

Modifications of allocated interventions are not considered.

### Strategies to improve adherence to interventions {11c}

Stimulation protocols for all interventions have been already established for their effectiveness in patients with PD [9, 44]. Participants will be informed that, regardless of their assigned study group, they could expect to experience several known benefits of rTMS. Furthermore, given that all interventions will occur within the hospital, clinical research coordinators will maintain periodic contact through phone calls or text messages to remind the participants of upcoming interventions.

### Relevant concomitant care permitted or prohibited during the trial {11d}

Participants are required to maintain a consistent dosage of their antiparkinsonian medication throughout the study. In addition, the same dose of physical therapy is permitted. Any changes are prohibited.

### Provisions for post-trial care {30}

In this study, participants will receive compensation through the clinical trial insurance coverage held by the investigator in adherence to the study’s victim compensation protocol. This coverage extends to physical damage incurred as a result of the investigational medical device and any adverse, unintended reactions arising from the study procedures. Nevertheless, compensation is not applicable in scenarios where the investigational medical device fails to yield valid results or prove beneficial, or in cases of damage resulting from negligence on the part of the participant, among other specified exclusions.

### Outcomes {12}

Pre-intervention evaluations will be conducted within 3 days before the initial intervention session. Post-intervention evaluations will be conducted within 48 h after the final intervention session. Follow-up evaluations will be conducted 2 months after the intervention concludes. The primary outcome is the difference in TUG time between the pre- and post-intervention evaluations. Secondary outcome variables include the TUG measured at follow-up evaluations. Additionally, the TUG-Cog, MDS-UPDRS Part III, New Freezing of Gait Questionnaire (NFoGQ), Digit Span, trail-making test, transcranial magnetic stimulation-induced motor-evoked potential (TMS-induced MEP), diffusion tensor imaging (DTI), and rsfMRI are assessed pre-intervention, post-intervention, and at follow-up to investigate the effectiveness of personalized rTMS.

The TUG assesses ambulatory functions, including gait, balance, mobility, and fall risk. In the TUG, the participants are instructed to rise from a chair, walk to a traffic cone located 3 m away at a comfortable pace, return to the chair, and sit down. The TUG-Cog involves the simultaneous execution of the TUG test and serial subtraction in three, starting from a randomly selected number between 80 and 100 [34]. The TUG-Cog is designed to provide a comprehensive assessment by incorporating both motor and cognitive tasks. The TUG and TUG-Cog are each measured twice, and the mean values are utilized for analysis. During the TUG and TUG-Cog, the participants wore shoes equipped with a smart insole (Gilon Gait Data Collector & Analyze MD, Gilon Inc., Gyeonggi, Republic of Korea). The smart insole measures gait parameters including stride length, step count, cadence, velocity, distance, swing ratio, and foot plantar pressure (heel/mid/toe). The stride lengths of the TUG and TUG-Cog are utilized to assign sub-studies and evaluate the efficacy of the intervention. During the mid-intervention evaluation, the TUG is conducted without smart insoles.

The MDS-UPDRS part III assesses the motor symptoms of PD using 18 items, with each item scored on a scale of 0 to 4 [46]. Higher scores indicate more severe symptoms. The NFoGQ consists of nine items designed to assess the severity of freezing of gait (FoG) and gait disturbance [47]. The NFoGQ has demonstrated reliability in measuring both the severity of FoG and its functional impact in patients with PD.

Cognitive function is assessed using the Digit Span and trail-making test. The Digit Span evaluates attention and working memory. Participants are instructed to repeat a sequence of numbers, either in the same order (forward) or in the reverse order (backward). The sequence starts with three numbers in the forward task and progresses to nine numbers, whereas the backward task involves sequences from two numbers up to eight numbers. The trail-making test evaluates cognitive functions, such as cognitive processing speed and executive function. In trail-making test A, the participant connects numbers from 1 to 15, while in trail-making test B, the task involves connecting eight numbers and seven letters (Monday to Sunday in Korean) in alternating ascending order. The examiner measures the time taken by the participants to complete the task.

Cortical excitability through TMS-induced MEP from the bilateral first dorsal interosseous muscles is utilized to evaluate the efficacy of the intervention. The intensity is set at 120% of the RMT at intervals of 5 s or more, and repeated 10 times. The average amplitude of the top five responses is measured and evaluated. Brain imaging data, including rsfMRI, DTI, and T1-weighted structural images, will be acquired using 3-T scanners (Philips Ingenia CX, Philips Elition, Siemens Magnetom Trio, and Siemens Magnetom Vida). rsfMRI will be utilized to extract brain networks based on their functional connectivity. Changes in brain network characteristics due to the intervention will be examined through connectivity strength, graph theory, and large-scale network analyses of global and local networks, as well as intrahemispheric and interhemispheric networks. During the resting-state scan, participants will be instructed to keep their eyes closed and remain motionless. A total of 180 whole-brain images will be collected at each session using the following metrics: 75 axial slices, slice thickness = 2 mm, no gap, matrix size = 112 × 112 or 124 × 124, and repetition time = 2000 ms. DTI will be employed to extract the integrity of major neural pathways and structural networks using fiber tractography and to examine changes in the characteristics of the integrity and networks due to the intervention. Each session will acquire more than 30 diffusion-weighted images with b = 1000 s/mm^2^, ensuring a minimum of 75 axial slices, a slice thickness = 2 mm, no gaps, and a matrix size of = 112 × 112 or 128 × 128. T1-weighted structural images will be used to determine the individual target positions in the DLPFC. The images will be acquired with a resolution and slice thickness of 1 mm or less, following the recommendations of the Neurophet software for 3D modeling-based target positioning.

All evaluations will be conducted in the “on” state, representing the peak effect of PD medication. Outcome measures will be assessed at pre-, post-intervention, and follow-up evaluations. Evaluation during the intervention will only proceed for the TUG, TUG-Cog, and TMS-induced MEP.

### Participant timeline {13}

**Fig. 1:**
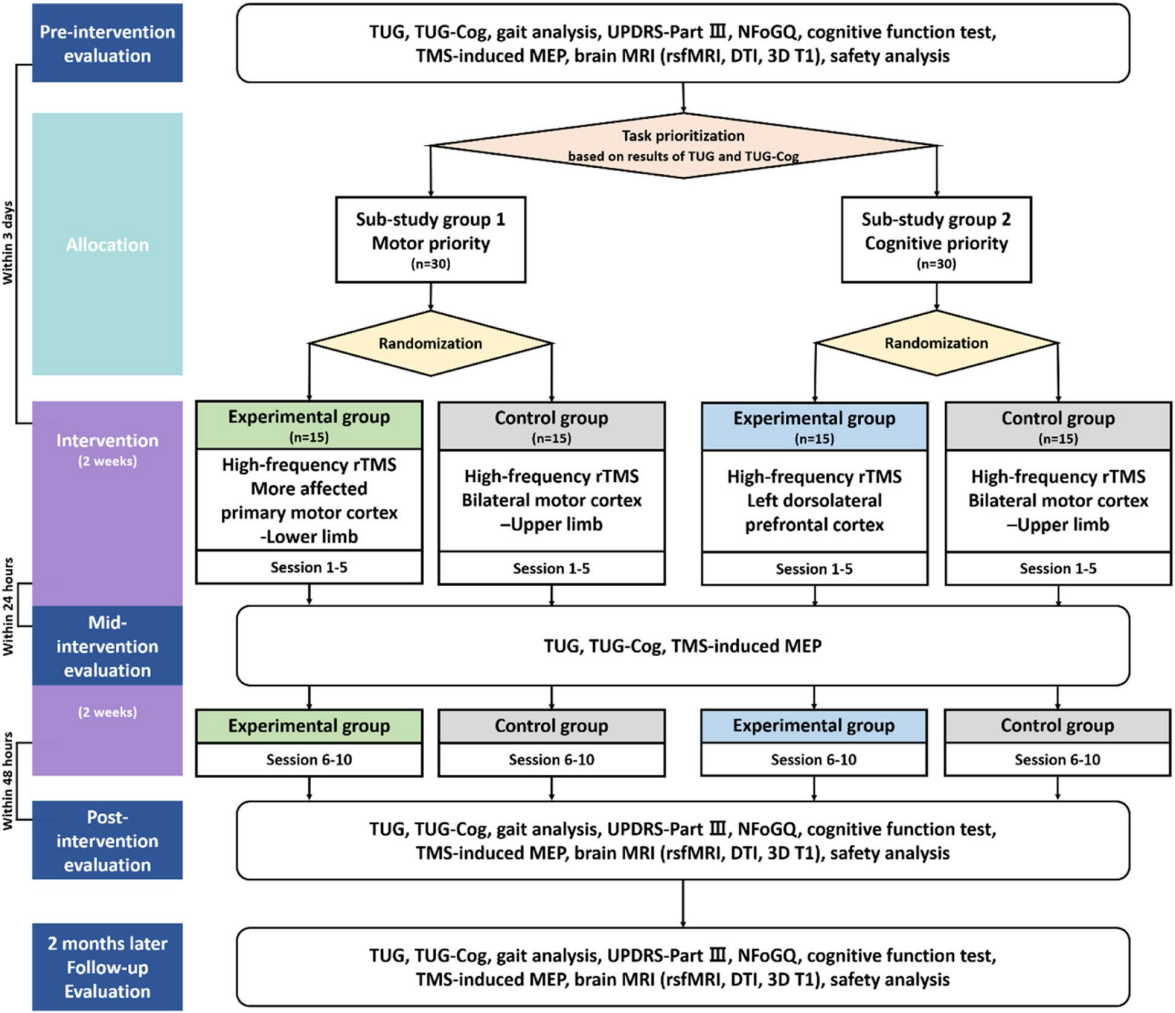
Flowchart through the entire study process *TUG* Timed-Up and Go, *TUG-Cog* Timed-Up and Go under cognitive dual-task condition, *UPDRS* Unified Parkinson’s Disease Rating Scale, *NFoGQ* New Freezing of Gait Questionnaire, *TMS-induced MEP* Transcranial magnetic stimulation-induced motor-evoked potential, *MRI* Magnetic resonance imaging, *rsfMRI* Resting state functional magnetic resonance imaging, *DTI* Diffusion tensor imaging

Figure 1 presents a comprehensive flowchart of the study process, including the allocation phase. After enrollment and screening, a pre-intervention evaluation is conducted. Based on the pre-intervention evaluation, participants are classified into sub-studies and allocated to either the experimental or control group. The first rTMS session takes place within 3 days of the pre-intervention evaluation. The initial 5 interventions are administered within 2 weeks, with a mid-intervention evaluation employed within 24 h following the fifth session. Subsequently, an additional 5 rTMS sessions are administered over the next 2 weeks, and within 48 h of completing all intervention sessions, an immediate post-intervention evaluation is conducted. Follow-up evaluation is performed 2 months after the completion of the intervention, marking the end of the study. The total study duration is anticipated to be approximately 12–14 weeks. The timelines for both the experimental and control groups in the sub-studies are identical (Table 2).

**Table 2:** Schedule of enrollment, interventions, and assessments in the study

	**Study period**						
	**Enrollment**	**Baseline**	**Intervention**			**Post-intervention**	**Close-out (2 months)**
**Timepoint**	*** − t***_***1***_	**t**_***0***_	***t***_***1–5***_	***t***_***6***_	***t***_***7–11***_	***t***_***12***_	***t***_***13***_
**Enrollment:**							
Informed consent	X						
Eligibility screen	X						
Allocation		X					
**Interventions:**							
* HF rTMS over M1-LL*			X		X		
* HF rTMS over DLPFC*			X		X		
* HF rTMS over bilateral M1-UL*			X		X		
**Assessments:**							
* TUG, TUG-Cog*		X		X		X	X
* Gait analysis*		X				X	X
* MDS-UPDRS Part III*		X				X	X
* NFoGQ*		X				X	X
* Cognitive function test*		X				X	X
* TMS-induced MEP*		X		X		X	X
* Brain MRI*		X				X	X
* Adverse event measure*		X	X	X	X	X	X

*HF rTMS* High-frequency repetitive transcranial magnetic stimulation, *M1-LL* Primary motor cortex in the lower limb, *DLPFC* Dorsolateral prefrontal cortex, *M1-UL* Primary motor cortex in the upper limb, *TUG* Timed-Up and Go, *TUG-Cog* Timed-Up and Gg under cognitive dual-task condition, *MDS-UPDRS* Movement Disorder Society-Unified Parkinson’s Disease Rating Scale, *NFoGQ* New Freezing of Gait Questionnaire, *TMS-induced MEP* Transcranial magnetic stimulation-induced motor-evoked potential, *MRI* Magnetic resonance imaging

### Sample size {14}

The primary outcome is the change in TUG time between pre- and post-intervention evaluations. The power of the study was set at 80%, with a significance level (α) of 5%. The clinically significant effect size (δ) was determined to be 4.9, and the expected standard deviation (σ) is estimated to be 4.0 [48, 49]. The analysis was conducted using Lehr’s formula, resulting in a required sample size of 10.6 [50].

$$\text {Lehr's}\,\mathrm{formula}=\left(16/(\delta/\sigma)\right)=\left(16/(4.9/4.0)\right)^2=10.6$$

The follow-up rate was targeted at 75%, based on the conventional outpatient rehabilitation treatment criteria over a 4-week period. Therefore, the sample size for each sub-study was determined to be 30.

### Recruitment {15}

Study participants will be recruited by posting notices on the bulletin boards of respective 5 hospitals. The investigators will not exclude potential participants based on race or socioeconomic status. If eligible according to the study criteria, every effort will be made to facilitate the participation of eligible patients in this research. Additionally, patients will be informed about the purpose of the study to ensure representation of the entire PD patient population receiving treatment at each institution.

## Assignment of interventions: allocation

### Sequence generation {16a}

Participants are randomly allocated to the experimental and control groups of each sub-study. A designated individual, who is not involved in this study, utilizes the www.randomization.com to generate a randomization table, ensuring a 1:1 allocation between the experimental and control groups before the enrollment of the first participant. The chief investigator (CI) maintains the confidentiality of the randomization table matching list and refrains from revealing it until the completion of the final statistical analysis, managing it securely as per protocol.

### Concealment mechanism {16b}

The order of assignment is concealed using an electronic data capture system until each participant is assigned.

### Implementation {16c}

The allocation sequence is generated by a third party who is not involved in the study. The physician investigators enroll the participants, and based on the results of the pre-intervention evaluation, the participants are assigned to the sub-studies. The rTMS administrator verifies the electronic data capture system to determine the participant’s assigned group.

### Assignment of interventions: blinding

#### Who will be blinded {17a}

This study is conducted as a single-blind clinical trial with a blinded observer; the rTMS administrator is aware of the participant’s assigned group, while both the participant and assessor are unaware of which treatment is being implemented. The evaluation is performed by an investigator who does not administer rTMS, to ensure that the assessor remains blinded.

### Procedure for unblinding if needed {17b}

This is a single-blind study; therefore, the assessors and participants will not be informed of the study arm. However, unblinding should be considered in cases of serious medical emergencies. In the event of a serious medical emergency, unblinding will be performed only if information regarding the stimulation protocol affects the participant’s treatment. Unblinded participants will not be permitted to continue within the study.

## Data collection and management

### Plans for assessment and collection of outcomes {18a}

This multi-center study is conducted across five tertiary hospitals in the Republic of Korea. To ensure robust data quality, researchers from all the institutions convened multiple meetings to discuss and establish standardized assessment methods. Following these discussions, comprehensive training sessions were conducted to ensure that the assessors were well-versed in standardized methodologies. Most of the assessments used in this study are validated for both reliability and validity. Additionally, we meticulously document and disseminate protocols to conduct research evaluations, allowing for the ongoing scrutiny of evaluation methods and data collection forms.

### Plans to promote participant retention and complete follow-up {18b}

Clinical research coordinators in each hospital will communicate with the participants via phone calls or text messages to ensure their awareness and participation in upcoming evaluations and interventions.

### Data management {19}

To ensure data quality, the CI selected the clinical research organization responsible for overseeing various aspects of data management. The contract encompasses tasks, such as developing an electronic case report form (eCRF) database and implementing data management protocols. The database system is tasked with query programming, performing range checks for data values, and managing data archiving. Additionally, the data management team is responsible for creating a data validation plan, overseeing query management, coding adverse events, and reconciling serious adverse events.

### Confidentiality {27}

In accordance with the ethical guidelines, personal information and research outcomes of participants will be documented on the designated eCRF without exposing personal details such as the obligation record number and name of the participant. Access to these records is restricted to registered researchers to ensure confidentiality. The identities of the participants will be kept confidential in all instances of research presentation or publication. Additionally, any research data, including imaging data and documents, will be stored in password-protected files in a secure, locked facility. Researchers are required to retain all clinical trial-related records and informed consent forms for a period of 3 years from the conclusion of the research (Bioethics and Safety Act of the Republic of Korea), and documents beyond this retention period will be disposed of in accordance with the regulations outlined in the Personal Information Protection Act of the Republic of Korea.

### Plans for collection, laboratory evaluation, and storage of biological specimens for genetic or molecular analysis in this trial/future use {33}

Not applicable. No biological specimens are collected.

## Statistical methods

### Statistical methods for primary and secondary outcomes {20a}

Demographic data are presented as means and standard deviations for continuous variables, while frequencies and percentages are used for categorical variables. Efficacy analysis are based on the assessment of the change from pre-intervention evaluation within each sub-study. To compare the baseline characteristics between the experimental and control groups in each sub-study, Student’s *t*-test for normally distributed variables or the Wilcoxon singed-rank test for non-normally distributed variables is employed. The Shapiro–Wilk test is used to examine the normal distribution of the variables.

To evaluate the effects of time, group, and the interaction of time with the group, we employ repeated-measures analysis of variance and repeated-measures analysis of covariance for variables exhibiting a normal distribution. Non-parametric variables are analyzed using a generalized estimating equation. Statistical significance is set at *P* < 0.05. A comparison between the sub-studies is not considered.

### Interim analyses {21b}

Not applicable. No formal interim analysis has been planned.

### Methods for additional analyses (e.g., subgroup analyses) {20b}

Not applicable. No subgroup analysis has been planned.

### Methods in analysis to handle protocol non-adherence and any statistical methods to handle missing data {20c}

All participants involved in this study and those undergoing intervention are included in the intention-to-treat (ITT) set. Safety analyses are conducted based on the ITT dataset. Participants in the ITT set who undergo the TUG test at both pre- and post-intervention evaluations are categorized as the full analysis set (FAS). Efficacy analysis will be based on the FAS. Those in the ITT set who successfully complete the study with no significant protocol violations are classified into the per-protocol (PP) set. Efficacy analyses within the PP set are conducted alongside the FAS. In the event of disparities between the FAS and PP analyses, the reasons behind such difference will be investigated. For missing values, data will be analyzed using the last-observation-carried-forward method, assuming that the most recent observation was obtained at that time point.

### Plans to give access to the full protocol, participant-level data and statistical code {31c}

Not applicable. The datasets analyzed during the current study and statistical code are available from the corresponding authors on reasonable request, as is the full protocol.

## Oversight and monitoring

### Composition of the coordinating center and trial steering committee {5d}

To monitor trial progress, a monthly meeting will be convened by the coordinating center, consisting of the CI and Principal Investigators (PIs) from the five clinical trial sites. There is no independent trial steering committee, but each site has a Human Research Protection Program (HRPP) and a Quality Assurance (QA) department. The HRPP is responsible for protecting the rights and welfare of participants. The QA department ensures that research complies with applicable regulations, ethical principles, institutional policies, and approved protocols by conducting internal audits, managing non-compliance issues, assisting with researcher training, and implementing quality improvement measures.

### Composition of the data monitoring committee, its role and reporting structure {21a}

The Data Monitoring Committee, consisting of the CI and monitoring agents from each hospital, conducts monitoring every 6 months, with additional irregular monitoring in the event of serious adverse events. The sponsor has played no role in the study’s design and not involved in the collection, analysis, interpretation of data, or writing of the manuscripts.

### Adverse event reporting and harms {22}

During the clinical trial, personnel record adverse reaction details, including symptoms, onset dates, and resolution dates, in an adverse reaction record form. Severity is assessed using a scale ranging from negligible to critical. Causality is evaluated for obvious relevance, probable relevance, suspected relevance, low relevance, lack of relevance, or indeterminable status. Interventions for medical devices are categorized as discontinuation, reduction, increase, no change in dosage, unknown, or not applicable. The results of the interventions, specifying the resolution or worsening of adverse reactions, are also managed.

When reporting the study results, the PI provides a comprehensive description and assessment of all symptoms that occurred during the clinical trial. In case of a serious adverse event, the PI reports it to the IRB to determine the continuation or discontinuation of the study. Critical incidents, such as death or life-threatening events, necessitate reporting to the Ministry of Food and Drug Safety of the Republic of Korea within 7 days. Additionally, events requiring hospitalization or an extension of hospitalization resulting in irreparable damage, severe disability, or dysfunction must be reported within 15 days. The results of the interventions are methodically recorded and managed according to recovery/resolution status, ongoing recovery/resolution, non-recovery/non-resolution, recovery with residual effects, death, or unknown outcomes.

### Frequency and plans for auditing trial conduct {23}

Regularly scheduled plans for auditing trials are not in place. However, audits may be conducted at any time by the Ministry of Food and Drug Safety of the Republic of Korea or by an internal auditing organization within the institution where the clinical trial is being conducted. The audit process will be independent of the investigators and sponsor.

### Plans for communicating important protocol amendments to relevant parties (e.g., trial participants, ethical committees) {25}

Regarding important protocol amendments, the CI at Samsung Medical Center informs the PIs at each institution conducting the clinical trial. The CI is obligated to report these amendments to the Ministry of Food and Drug Safety of the Republic of Korea and the IRB of Samsung Medical Center. The PIs at each institution report these amendments to their respective IRBs. Additionally, PIs inform their research teams in detail about significant protocol modifications, and if necessary, notify the research participants.

### Dissemination plans {31a}

The trial has been registered at ClinicalTrials.gov (NCT06350617) and we will continuously update the trial status on the site throughout the study. The publication of academic papers is scheduled within 2 years of the completion of all data collection.

## Discussion

This study delineates a novel investigation into the administration of personalized rTMS aimed at enhancing ambulatory function by reinforcing the preserved functional reserves in patients with PD. Based on the assessments of patient functional capacity through single- and dual-task assessments, this study aims to classify patients with PD into distinct groups according to their motor or cognitive functional reserves, with the subsequent application of rTMS targeted at specific cerebral sites. The principal objective of both the motor and cognitive priority groups is to improve of ambulatory function. In the motor priority group, the goal is achieved by enhancing motor capacity through the application of high-frequency rTMS to the M1-LL. In contrast, in the cognitive priority group, a compensatory enhancement of ambulatory function is sought by strengthening cognitive abilities facilitated by the application of high-frequency rTMS to the left DLPFC.

This study has several limitations. First, as the study is conducted across five tertiary hospitals, two different rTMS stimulators are employed, which could potentially introduce variability in the stimulation parameters and outcomes. Second, the lack of a gold standard to determine functional priorities poses a challenge in precisely categorizing patients, potentially affecting the specificity and applicability of rTMS protocol tailored to individual needs. Nevertheless, the DTE battery based on TUG and TUG-Cog has established validity and reliability [34]. Additionally, both the TUG and TUG-Cog can be easily administered without the need for specialized equipment or tools, making them feasible for widespread use in clinical settings.

In conclusion, this study will reveal the effect of personalized rTMS compared to the conventional rTMS approach in PD. Furthermore, the findings of this study may provide empirical evidence for high-frequency rTMS protocol tailored to individual functional reserves to enhance ambulatory function in patients with PD.

### Trial status

- Protocol version: 1.2 (26 JAN 2024).
- Study start: 20 FEB 2024 (actual).
- Study completion: 31 DEC 2025 (estimated).

### Supplementary Information

Supplementary Material 1.

## Data Availability

Not applicable. Any data required to support the protocol can be supplied from the corresponding authors on request.

## Abbreviations

CI: Chief investigator
DAT: Dopamine transporter
DLPFC: Dorsolateral prefrontal cortex
DTE: Dual-task effect
DTI: Diffusion tensor imaging
eCRF: Electronic Case Report Form
IRB: Institutional Review Board
M1: Primary motor cortex
M1-LL: Primary motor cortex in the lower limb
M1-UL: Primary motor cortex in the upper limb
MDS-UPDRS: Movement Disorder Society-Unified Parkinson’s Disease Rating Scale
MRI: Magnetic resonance imaging
NFoGQ: New Freezing of Gait Questionnaire
PD: Parkinson’s disease
PI: Principal investigator
RMT: Resting motor threshold
rsfMRI: Resting state functional magnetic resonance imaging
rTMS: Repetitive transcranial direct magnetic stimulation
TMS-induced MEP: Transcranial direct magnetic stimulation-induced motor evoked potential
TUG: Timed-Up and Go
TUG-Cog: Timed-Up and Go under cognitive dual-task condition
